# Structure of a Membrane-Embedded Prenyltransferase Homologous to UBIAD1

**DOI:** 10.1371/journal.pbio.1001911

**Published:** 2014-07-22

**Authors:** Hua Huang, Elena J. Levin, Shian Liu, Yonghong Bai, Steve W. Lockless, Ming Zhou

**Affiliations:** 1 Verna and Marrs McLean Department of Biochemistry and Molecular Biology, Baylor College of Medicine, Houston, Texas, United States of America; 2 Department of Biology, Texas A&M University, College Station, Texas, United States of America; 3 Texas A&M Institute for Neuroscience, Texas A&M University, College Station, Texas, United States of America; University of Zurich, Switzerland

## Abstract

Membrane-embedded prenyltransferases from the UbiA family catalyze the Mg^2+^-dependent transfer of a hydrophobic polyprenyl chain onto a variety of acceptor molecules and are involved in the synthesis of molecules that mediate electron transport, including Vitamin K and Coenzyme Q. In humans, missense mutations to the protein UbiA prenyltransferase domain-containing 1 (UBIAD1) are responsible for Schnyder crystalline corneal dystrophy, which is a genetic disease that causes blindness. Mechanistic understanding of this family of enzymes has been hampered by a lack of three-dimensional structures. We have solved structures of a UBIAD1 homolog from *Archaeoglobus fulgidus*, AfUbiA, in an unliganded form and bound to Mg^2+^ and two different isoprenyl diphosphates. Functional assays on MenA, a UbiA family member from *E. coli*, verified the importance of residues involved in Mg^2+^ and substrate binding. The structural and functional studies led us to propose a mechanism for the prenyl transfer reaction. Disease-causing mutations in UBIAD1 are clustered around the active site in AfUbiA, suggesting the mechanism of catalysis is conserved between the two homologs.

## Abstract 2

A crystal structure of a member of the UbiA family of membrane-embedded prenyltransferases reveals the architecture of the active site and suggests a possible mechanism for catalysis.

## Author Summary

The biosynthesis of Vitamin K and Coenzyme Q requires the transfer of a long, hydrophobic moiety known as an isoprenyl onto an aromatic acceptor compound. This process is catalyzed by a family of proteins known as the UbiA proteins, which are embedded in the hydrophobic environment of cell membranes. To understand how the prenyltransfer reaction is carried out, we solved the three-dimensional structure of a member of the UbiA family by X-ray crystallography. This structure reveals how magnesium ions and the prenyl substrate are bound within a sealed amphipathic chamber inside the protein and suggests how the reaction intermediate may be stabilized by the protein and protected from the solvent. Functional studies carried out on another member of the UbiA family, as well as comparison to known disease-causing mutations in the human homolog UBIAD1, demonstrate that the residues involved in this process are conserved across the UbiA family.

## Introduction

Vitamin K is an essential cofactor required for the posttranslational modification of proteins involved in blood-clotting and normal bone metabolism. One of the major forms of vitamin K in humans, menaquinone-4, is produced by cleaving the phytyl group from dietary phylloquinone to produce menadione, which is then modified with a polyprenyl group donated from geranylgeranyl diphosphate (Figure S1). This latter step is catalyzed by the protein UBIAD1, a member of a family of integral membrane proteins known collectively as UbiA prenyltransferases [1]–[3]. Recently, it has also been proposed that UBIAD1 is responsible for the prenylation of coenzyme Q10 in Golgi membranes [4]. Missense mutations to the UBIAD1 gene are the underlying cause of the genetic disorder Schnyder corneal dystrophy (SCD), which causes accumulation of cholesterol and phospholipids in the cornea of the eye, eventually leading to blindness [5].

**Figure 1:**
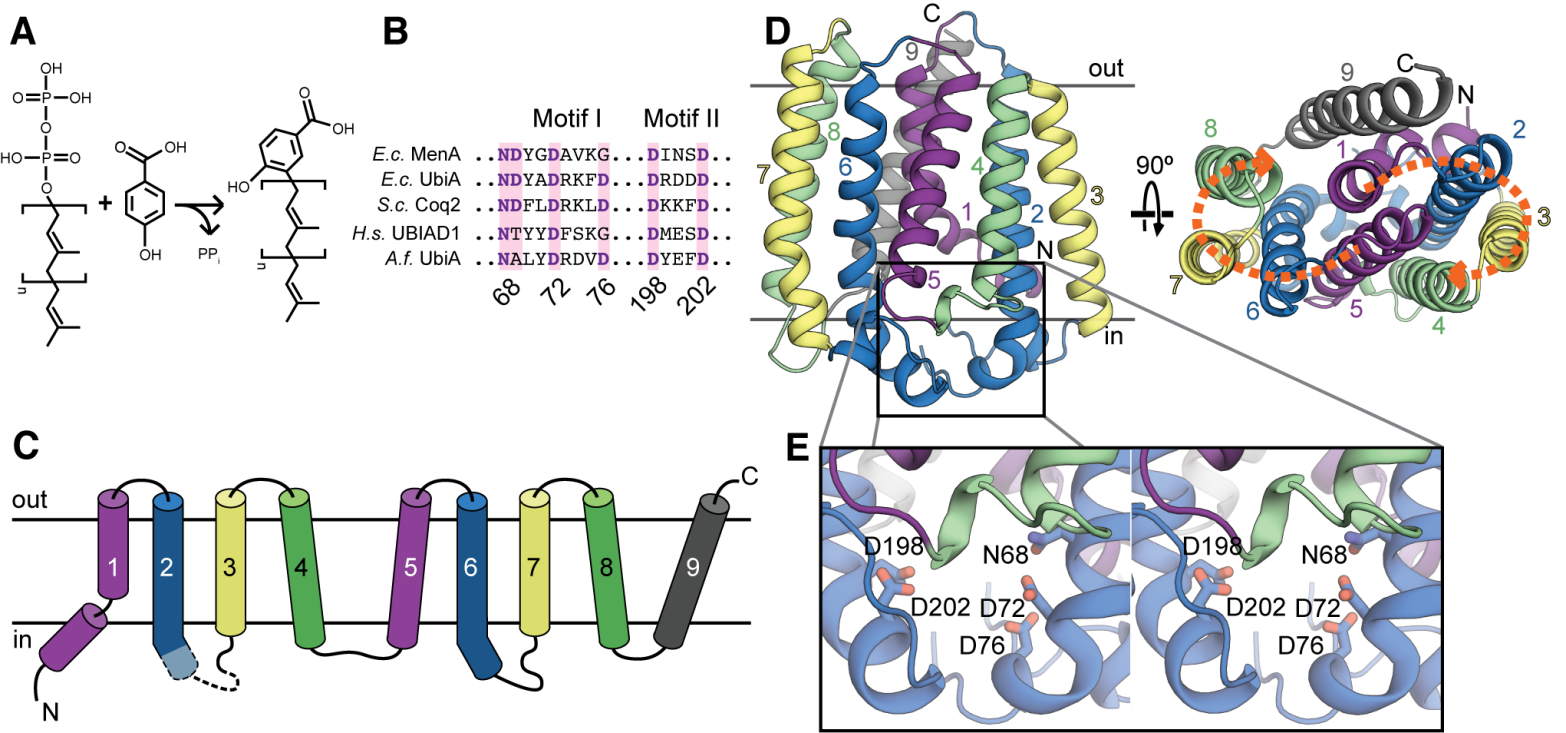
The UbiA fold. (A) A representative reaction catalyzed by UbiA family members. Polyprenyl is transferred from diphosphate to 4HB to form 3-polyprenyl-4-hydroxybenzoate, a precursor to ubiquinone. The square brackets denote a single five-carbon prenyl unit. (B) The two conserved aspartate-rich motifs characteristic of the UbiA family. Residue numbers correspond to the *Archaeoglobus fulgidus* UbiA sequence. Ec, *Escherichia coli*; Sc, *Saccharomyces cerevisiae*; Hs, *Homo sapiens*; Af, *Archaeoglobus fulgidus*. (C) Topology diagram of AfUbiA, with the transmembrane helices colored in pairs of equivalent helices in the four-helix bundles. Dashed lines indicate the region of L2–3 that is disordered in the SeMet crystal structure. (D) Cartoon representation of the AfUbiA structure viewed from within the plane of the membrane (left) and from the extracellular side of the membrane (right). The transmembrane helices are colored according to the same scheme as in panel (C). Orange arrows indicate the two pseudosymmetric bundles. (E) Magnified stereo view of the boxed area in panel (D), showing residues from aspartate-rich Motif I and II as sticks.

Membrane-embedded prenyltransferases belonging to the UbiA family are found in every branch of life, and are involved in the biosynthesis of a highly diverse range of molecules, including respiratory lipoquinones such as ubiquinone and menaquinone [6]–[8], prenylated hemes and chlorophylls [9],[10], archaeal lipids [11], numerous prenylated plant flavonoids [12], the antibiotic aurachin [13], Vitamin E [14],[15], and bacterial cell wall precursors [16]. Although the nature of the prenyl acceptor and donor vary considerably, reactions catalyzed by UbiA homologs are Mg^2+^-dependent and generate pyrophosphate as a leaving group. A representative reaction, catalyzed by the eponymous *E. coli* protein UbiA, involves the cleavage of the C–O bond in polyprenyl diphosphates of variable length and transfer of the prenyl chain to the *ortho* position of the phenol 4-hydroxybenzoic acid (4HB; Figure 1A). UbiA family members are typically predicted to contain eight or nine transmembrane helices and have two characteristic conserved motifs with the consensus sequences NDXXDXXXD and DXXXD (Figure 1B), often referred to as the first and second aspartate-rich motifs, respectively.

To understand the structural basis of UbiA function, we set out to elucidate the structure of a member of the UbiA family using X-ray crystallography. The resulting structures of an archaeal homolog reveal locations of Mg^2+^ and polyprenyl diphosphate binding sites, a possible hydrophobic substrate tunnel allowing the protein to accommodate polyprenyls of variable length, and the location of a cluster of highly conserved residues forming a potential catalytic site.

## Results

### Fold of AfUbiA

After screening a large number of bacterial and archaeal UbiA proteins for suitability for crystallization, a homolog from the extremophile *Archaeoglobus fulgidus* (AfUbiA) was chosen for further study based on its stability in detergent (Figure S2A). Crystals of the selenomethionine-substituted protein diffracted to 3.2 Å, and the structure was solved by single-wavelength anomalous dispersion (Table S1). Despite the modest resolution, assignment of the sequence register was greatly facilitated by the high quality of the experimentally phased electron density maps, and by the locations of five selenomethionine residues (Figure S2B,C). The selenomethionine structure was then used as a molecular replacement search model for 2.4 and 2.5 Å datasets collected on native AfUbiA crystals, grown in lipidic cubic phase (LCP) and soaked with either geranyl diphosphate (GPP) or dimethylallyl diphosphate (DMAPP) prior to freezing (Table S1, Figure S2D). The final models for the two substrate-bound structures contain four AfUbiA molecules in the asymmetric unit, with one molecule of GPP or DMAPP and two Mg^2+^ per protein chain. Comparison of the detergent and LCP crystal forms shows that none of the interaction surfaces between neighboring protomers are conserved between the different lattices, and so the protein is likely a monomer in the membrane (Figure S2E).

The AfUbiA structure contains a total of nine transmembrane helices, with the N and C termini emerging on opposite sides of the membrane (Figure 1C). Based on the distribution of positive and negative charges in the soluble loops, the N terminus of the protein is probably oriented towards the cytoplasm and the C terminus to the extracellular side [17]. This assignment is also consistent with the experimentally determined orientation of *E. coli* UbiA [18]. The first eight helices can be grouped into two bundles of four helices each (Figure 1D). The loops connecting the transmembrane helices are short, with the exception of the cytoplasmic loops connecting TM2 and 3 (L2–3) and TM6 and 7 (L6–7), which are both over 25 and 18 residues in length, respectively, and contain short helical regions. The two conserved aspartate-rich motifs are both positioned on the cytoplasmic side of the protein, between the C-terminal ends of TM2 and TM6 and the L2–3 and L6–7 loops (Figure 1E). Interestingly, the conserved motifs as well as the large cytoplasmic loops that follow them are at equivalent positions in the two four-helix bundles. Closer examination of the two bundles reveals that they are structurally homologous and can be superposed by a twofold pseudosymmetry axis running through the center of the protein perpendicular to the bilayer (Figure 1D, Figure S3). This raises the possibility that the UbiA fold may have arisen from the duplication of an ancient four-helix, dimeric protein.

The crystal structure of another archaeal UbiA homolog from *Aeropyrum pernix* (ApUbiA) was recently reported with a resolution of 3.6 Å [19]. The overall fold of AfUbiA is similar to that seen in ApUbiA, but there are differences in the location and coordination of Mg^2+^ and substrate (detailed in the section “Differences between the AfUbiA and ApUbiA crystal structures”). Both UbiA family members resemble proteins belonging to the isoprenoid synthase superfamily [20], and in particular, those members that catalyze the synthesis of all-trans polyprenyls by repeated addition of isopentenyl pyrophosphate (IPP; Figure S1), the trans-IPPSs. These enzymes are also Mg^2+^-dependent and contain similar aspartate-rich motifs. Although any sequence identity between the soluble and transmembrane families is negligible, comparison of AfUbiA to the trans-IPPS farnesyl diphosphate synthase (FPPS) from *E. coli* (Figure S4A–B) [21] reveals that the two four-helix bundles comprising helices 1–8 in AfUbiA and 2–9 in FPPS are superposable. TM9 in AfUbiA and the first helix in the trans-IPPS fold have no equivalent in the other family. Nevertheless, despite their structural similarity, examination of the distribution of charged residues on the two enzymes clearly reveals their distinct identities as soluble and transmembrane proteins (Figure S4C–D).

### Architecture of the Central Cavity and Substrate-Binding Site

**Figure 2:**
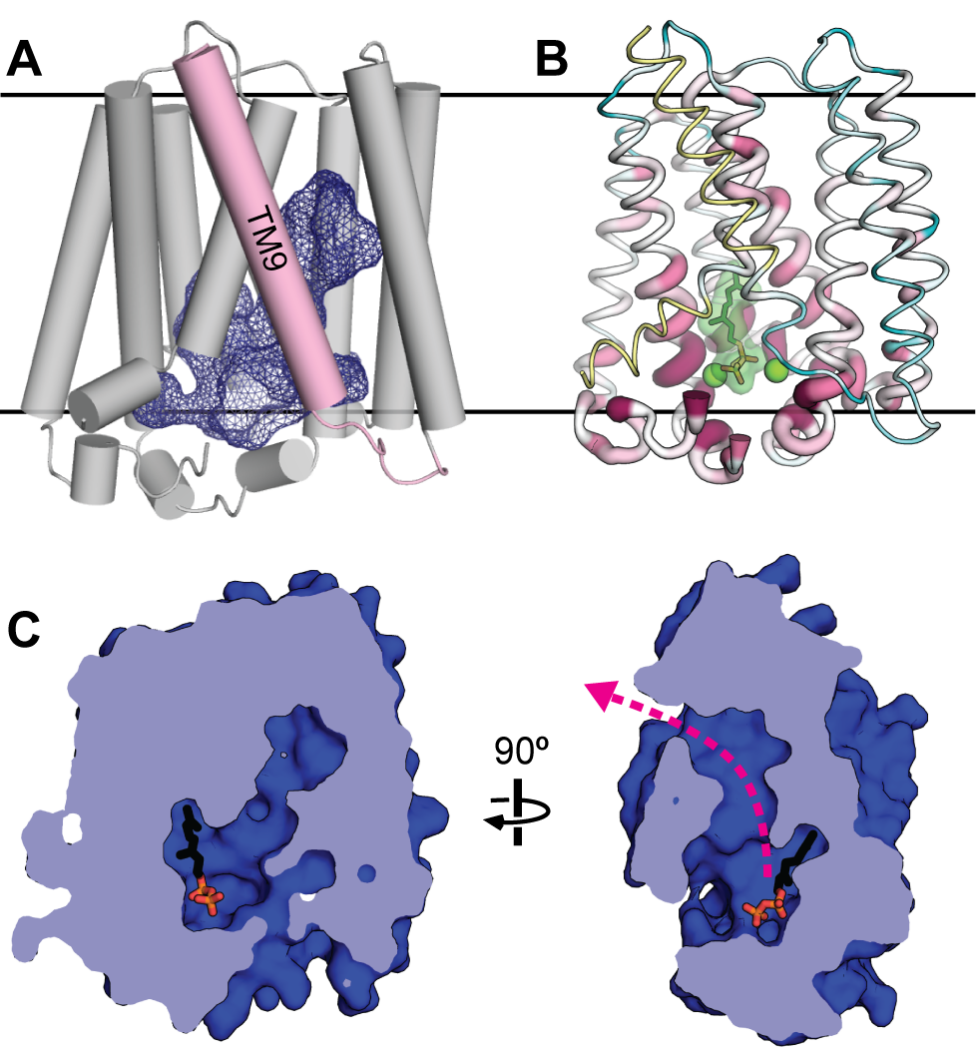
The substrate-binding cavity and possible substrate tunnel. (A) The solvent-accessible surface of the central cavity in the GPP-bound AfUbiA structure is shown as a blue mesh. Helix TM9 is highlighted in pink. (B) A ribbon representation of the AfUbiA structure in which the thickness and color of the ribbon indicate the degree of conservation. Highly conserved residues are thicker and colored dark purple; poorly conserved residues are thinner and colored teal. Residues colored yellow were not included in the multiple sequence alignment used to calculate the conservation scores. GPP and Mg^2+^ are shown in green. (C) Cutaway view of the central cavity and putative substrate tunnel from two perpendicular directions in the plane of the membrane, with the extracellular side on top. The molecule of GPP is shown as sticks. The pink arrow indicates a hydrophobic tunnel that opens into the membrane bilayer.

The bound isoprenyl-diphosphates and Mg^2+^ are located in a large cavity at the interface between the four-helix bundles near the cytoplasmic side, which is partly closed off from the solvent by the L2–3 and L6–7 loops (Figure 2A). Interestingly, in the unliganded structure, a 13-residue long region of the L2–3 loop is disordered, leaving this cavity widely accessible to the solvent, whereas all but four residues become resolved in the GPP-bound structure. In the DMAPP-bound structure, the entire loop is resolved and completely occludes the cavity from the solvent (Figure S5A,B). This difference may be attributed to the lower resolution of the unliganded structure, however, as the substrate in the DMAPP-bound structure is occluded from the cytoplasm, a more likely possibility is that substrate binding induces conformational changes in the L2–3 loop and thus seals off the active site.

Near the cytoplasm, the central cavity is broad and is lined with polar and charged residues, including the aspartate-rich motifs and many of the other residues that are most conserved across the UbiA family (Figure 2B). The cavity becomes more hydrophobic and tapers into a narrow tunnel as it extends deeper into the transmembrane region of the protein. Approximately halfway into the bilayer, the tunnel bends sharply and forms a fenestration in the side of the protein that opens into the bilayer (Figure 2C). This tunnel could offer a possible explanation for how UbiA family members utilize prenyl donors of varying lengths, which range from DMAPP (C_5_) [22] to dodecaprenyl phosphate (C_60_) [16]. The latter substrate approaches 60 Å in length in a fully extended conformation; in comparison, the membrane-spanning region of AfUbiA is less than 40 Å. The hydrophobic tunnel in AfUbiA could potentially accommodate up to six prenyl units, and even longer polyprenyls could bind to the protein by extending directly into the hydrophobic core of the bilayer.

**Figure 3:**
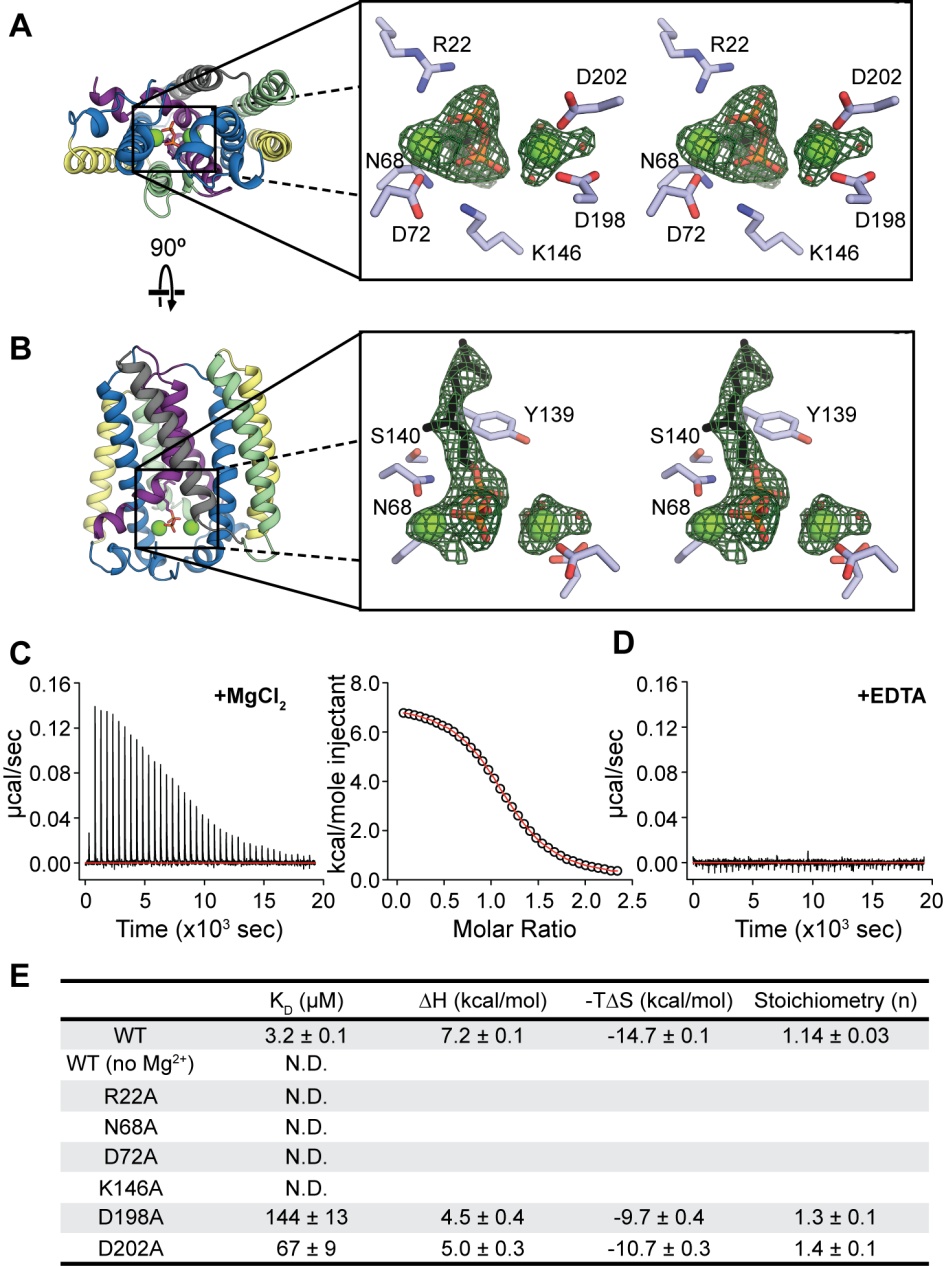
Substrate binding and active site. (A) Stereo view of the GPP binding site, viewed from the cytoplasmic side of the membrane. Two Mg^2+^ atoms (green spheres) and a GPP molecule are shown in the binding site. Residues that potentially bind to Mg^2+^ and the diphosphate are labeled. (B) Stereo view of the active site from within the plane of the membrane. Conserved residues proposed to stabilize the intermediate state are labeled. The green mesh in both figures corresponds to F_o_-F_c_ density contoured at 3.0 σ. (C and D) Binding of GPP to detergent-solubilized AfUbiA measured by ITC. Heats from successive injections of GPP were measured in the presence of 2 mM MgCl_2_ (C) or 1 mM EDTA (D). Right panel in (C) shows the fit to a one-site model. (E) Table of thermodynamic values for GPP binding to WT and mutant AfUbiA measured by ITC. K_D_, ΔH, and n were obtained by fitting a binding isotherm described in the Methods section. The thermodynamic relation ΔG = ΔH−TΔS was used to calculate −TΔS at 25°C with errors propagated. “N.D.” indicates no binding detected. Each value is the mean and s.e.m. of three ITC experiments.

In the GPP-bound structure, the substrate is located in the central cavity with its diphosphate positioned between the two aspartate-rich motifs (Figure 3A,B). Two electron densities are also visible on either side of the diphosphate that likely correspond to Mg^2+^. This observation is consistent with data showing that the activity of UbiA family members is Mg^2+^-dependent, as well as extensive mutagenesis experiments, confirming the importance of the two motifs for activity in *E. coli* UbiA [23] and other homologs [13],[24]. The two Mg^2+^ are coordinated by N68 and D72 in the first aspartate-rich motif and D198 and D202 in the second aspartate-rich motif (Figure 3A). The conserved aspartate D76 is too far away to bind directly to Mg^2+^ in the first motif, but could interact indirectly by stabilizing a water molecule coordinating the ion. The diphosphate group of GPP is stabilized by Mg^2+^ in the first motif, which bridges two oxygens with coordination distances of 2.3 and 2.6 Å. In contrast to the first motif, the Mg^2+^ bound to the second motif is 3.5–4.0 Å away from the diphosphate, which is significantly farther than the expected coordination distance of 2.0–2.3 Å. Additional interactions between the protein and GPP oxygens are provided by the basic residues R22 and K146. The GPP molecule is slightly kinked after the diphosphate, so that the isoprenyl tail extends along the wall of the cavity close to highly conserved residues on TM2, 4, and 5. In particular, the C–O bond cleaved in the prenyltransfer reaction is positioned near a cluster of conserved polar residues including N68, Y139, and S140 (Figure 3B). The geometry of the Mg^2+^ and substrate binding sites in the DMAPP-bound structure is similar (Figure S5C,D).

We used two approaches to verify the interactions between the bound substrate, ions, and protein in our crystal structure. First, to confirm the Mg^2+^ binding sites, we co-crystallized the protein with Cd^2+^ (Table S1). Two strong electron densities consistent with Cd^2+^ appear coordinated by the aspartate-rich motifs, which align well to the Mg^2+^ locations in the GPP-bound structure (Figure S6). Second, we used isothermal titration calorimetry (ITC) to measure the Mg^2+^ dependence of GPP binding to AfUbiA. In the presence of 2 mM MgCl_2_, GPP binds to AfUbiA with a K_D_ of 3.2±0.1 µM (Figure 3C). However, when 1 mM EDTA was added instead of MgCl_2_, no GPP binding was observed (Figure 3D). Residues N68, D72, D198, and D202 from the two aspartate-rich motifs, as well as the basic residues R22 and K146, were then mutated to alanine to test their contribution to Mg^2+^-dependent GPP binding (Figure 3E, Figure S7). Mutations to all six residues had pronounced effects on binding of GPP to AfUbiA. Four of the mutations completely abolished GPP binding, whereas the effects of the D198A and D202A mutations were comparatively mild, increasing the K_D_ by 45- and 21-fold, respectively. This is consistent with the observation that the distance between the Mg^2+^ bound to the second aspartate-rich motif and the GPP diphosphate is outside of the typical coordination distance range.

### Differences Between the AfUbiA and ApUbiA Crystal Structures

There are notable differences in the shape of the substrate-binding cavity and in the organization of the active sites of the AfUbiA and ApUbiA structures. The central cavity in ApUbiA is smaller than in AfUbiA, largely because ApUbiA lacks the long hydrophobic tunnel and second opening observed in AfUbiA. For the ApUbiA structure, it was proposed that longer prenyl chains may extend out of the protein via its single entrance to the central cavity, which is closer to the membrane interface than in AfUbiA. This mechanism of accommodating long prenyl chains is not likely for the current AfUbiA structure, because although the cytoplasmic opening exists in unliganded AfUbiA, it is completely closed in the DMAPP-bound structure (Figure S5B). 

**Figure 4:**
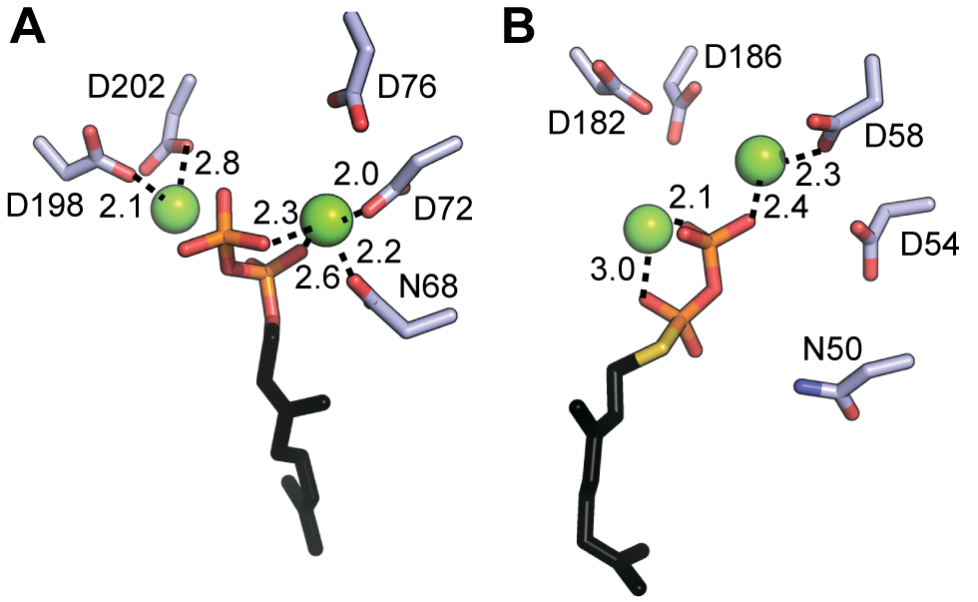
Comparison of the AfUbiA and ApUbiA active sites. (A and B) Interactions between Mg^2+^, GPP/GSPP, and residues in the first and second aspartate-rich motifs are shown for (A) AfUbiA and (B) ApUbiA (PDB accession 4OD5). All interaction distances of 3.0 Å or less are marked.

In both structures, the bound Mg^2+^ and the diphosphate moiety are located in the central cavity between the two conserved aspartate-rich motifs; however, interactions between the protein and ligands are different (Figure 4). Although two bound Mg^2+^ were modeled in both structures, in the current AfUbiA structure, N68, D72, D198, and D202 directly coordinate the Mg^2+^, while in the ApUbiA structure all the corresponding residues are >3.4 Å away from Mg^2+^. These four residues were demonstrated to be important for Mg^2+^-dependent GPP binding according to our ITC data (Figure 3E). The differences in Mg^2+^ location and coordination between the two structures are likely attributable to the significantly lower resolution (3.6 Å) of the ApUbiA structure.

4HB was also modeled into the ApUbiA structure, although there is currently no direct biochemical evidence that it can act as a prenyl acceptor for ApUbiA. This proposed 4HB binding site is unlikely to hold the prenyl acceptor in the current AfUbiA structure, as its position clashes with the location of the geranyl moiety. We were also unable to detect binding of 4HB to AfUbiA by ITC.

### Conservation with Other UbiA Family Members and Implications for the Catalytic Mechanism

**Figure 5:**
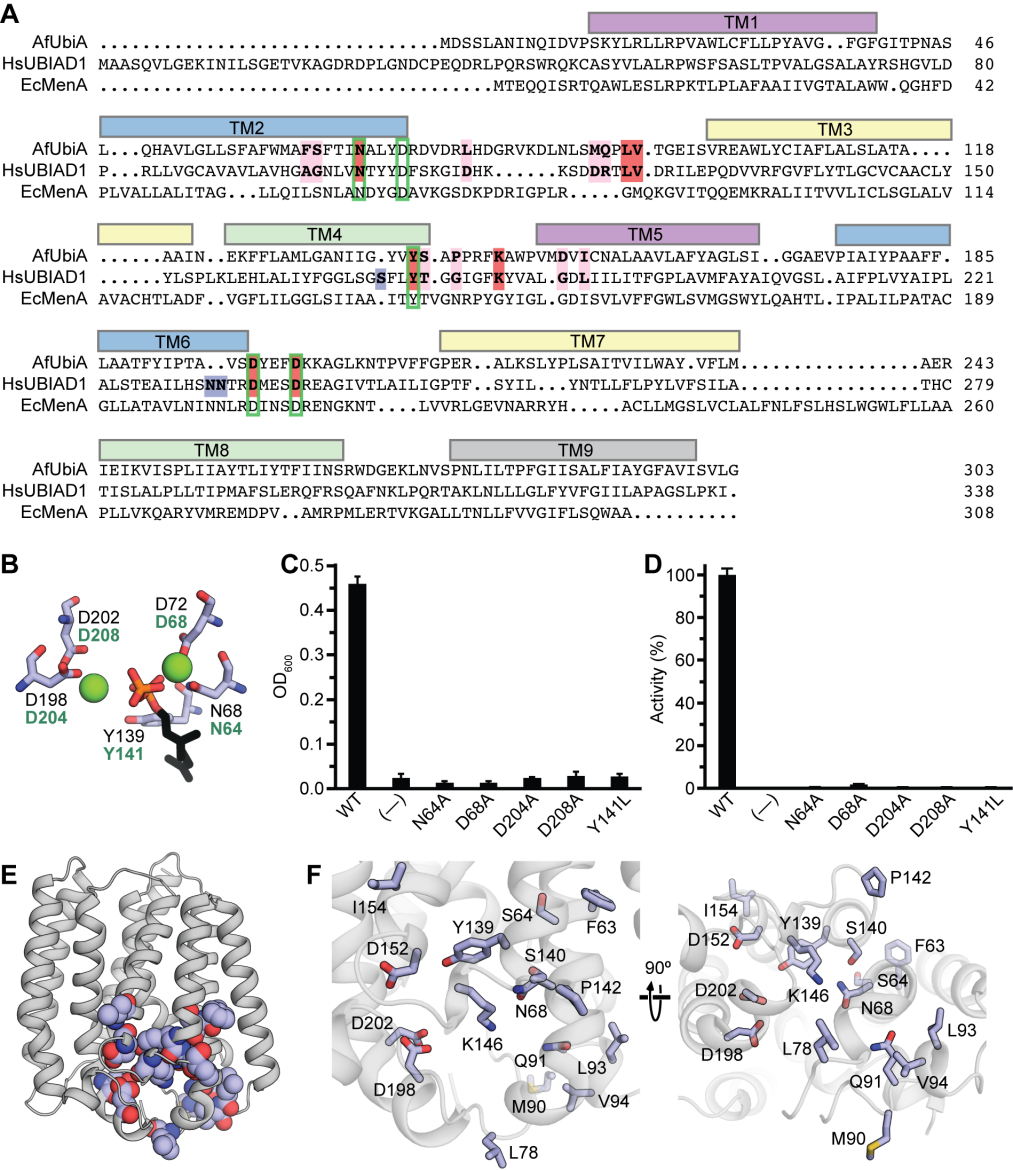
Conservation of active site residues across the UbiA family. (A) A sequence alignment of AfUbiA, human UBIAD1, and EcMenA. Residues currently known to be mutated in SCD patients are highlighted in pink, red, or blue in the AfUbiA and HsUBIAD1 sequences. Red indicates mutated residues that are identical between the two proteins; blue indicates residues that align with gaps in the AfUbiA sequence. Green boxes indicate locations of residues that were mutated in the EcMenA functional assays. The colored bars above the alignment indicate locations of transmembrane helices in the AfUbiA crystal structure. (B) The locations of the active site residues marked with green boxes in panel (A) are shown relative to bound Mg^2+^ and GPP in the AfUbiA structure. Black labels correspond to residue numbers in the AfUbiA structure, green labels to the equivalent residues in EcMenA. (C) A *menA* ^−^ *E. coli* strain was transformed with plasmids containing either WT or mutant EcMenA, and grown in suspension cultures in an anaerobic chamber. The optical densities at 600 nm were measured after 24 h. Cells transformed with an unrelated protein (TrkH) were used as the negative control. Error bars are standard deviations of three experiments. Data used to calculate the bar graphs are shown in Table S2. (D) Membranes were purified from *E. coli* overexpressing WT or mutant EcMenA and incubated at 37°C for 10 min with 2 mM DHNA, 1 mM GPP, and 5 mM MgCl_2_. Product formation was measured by HPLC and is shown as a percentage of the activity for WT EcMenA. Membranes from cells overexpressing EcUbiA, which is selective for 4HB as the prenyl acceptor, were used as a negative control. Error bars are standard deviations of three experiments. Data used to calculate the bar graphs are shown in Table S3. (E) Residues currently known to be mutated in SCD patients are shown as spheres on the structure of AfUbiA. (F) The same residues are shown as sticks in a closer view of the substrate-binding cavity from within the plane of the membrane (left) and from the intracellular side (right).

Although two crystal structures are available now for the UbiA family of proteins, both AfUbiA and ApUbiA are from archaeal thermophiles and enzymatic activity has not been demonstrated for either of the proteins. To understand the relevance of the AfUbiA structure to other UbiA family members, we mutated a number of residues on the *E. coli* MenA homolog (EcMenA) that are equivalent to key active site residues in AfUbiA (Figure 5A, Figure 5B). EcMenA catalyzes the transfer of a prenyl chain onto 1,4-dihydroxy 2-naphthoic acid (DHNA) to produce the menaquinone precursor demethylmenaquinone. Two independent functional assays were used to measure the effects of mutations on EcMenA: an *in vivo* genetic complementation assay in which growth under anaerobic conditions was measured in an *menA* ^−^ *E. coli* strain [25] transformed with WT or mutant EcMenA (Figure 5C), and an *in vitro* assay measuring prenyltransferase activity with purified membranes from *E. coli* cells overexpressing WT or mutant EcMenA (Figure 5D, Figure S8). For both assays, mutations to the equivalents of N68 and D72 in the first aspartate-rich motif and D198 and D202 in the second motif resulted in total or near-total loss of function. Mutation of the highly conserved tyrosine (Y139 in AfUbiA) near the C–O bond also resulted in loss of function.

Functional data for eukaryotic UbiA homologs are currently scarce, but missense mutations to 19 different residues on human UBIAD1 are known to cause SCD [5],[26]–[35]. Of these, three align to insertions not present on AfUbiA (Figure 5A). As shown in Figure 5E, the remaining 16 mutated residues all map to the region around the putative active site at the cytoplasmic end of the cavity. The residues Y174 and T175 on human UBIAD1 are equivalent to Y139 and S140, which belong to the cluster of polar residues on TM2 and TM4 likely important for catalysis; the residues A97 and G98 (F63 and S64 on AfUbiA) pack into the interface between TM2 and TM4 near this site (Figure 5F). Residues N102, K181, D236, and D240 on UBIAD1 are homologous to N68, K146, D198, and D202, which form part of the Mg^2+^/diphosphate binding site (Figure 3A). Residues 112, 118, 119, 121, and 122 on UBIAD1 align to residues 78, 90, 91, 93, and 94 on the highly mobile L2–3 loop of AfUbiA, which changes conformation upon substrate binding in AfUbiA. Potential functions for G177, G186, and L188 (P142, D152, and I154 on AfUbiA) are less evident, but all three residues are located in close proximity to the proposed active site.

**Figure 6:**
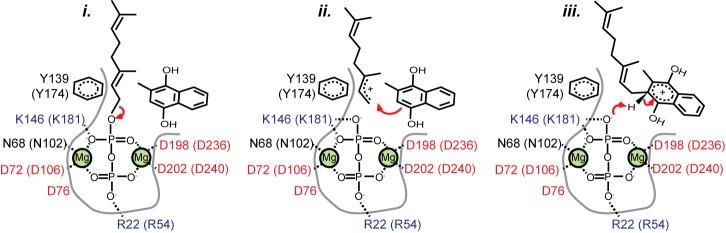
The proposed UbiA prenyltransferase reaction mechanism. Schematic showing a potential three-stage ionization (*i*), condensation (*ii*), and elimination (*iii*) reaction mechanism for prenyl transfer, with key conserved residues on the enzyme highlighted. The prenyl acceptor shown is the proposed substrate for UBIAD1, the reduced form of menadione (2-methyl-1,4-dihydroxynaphthoquinol); residue numbers are for AfUbiA and human UBIAD1 (in parentheses).

Overall, the above experiments on EcMenA and the mapping of UBIAD1 mutations onto the AfUbiA structure suggest that the fold and location of substrate-binding sites are conserved across the UbiA family, and that the mechanism of the prenyltransfer reaction is conserved as well. Although this mechanism is currently unknown, possible clues may be found by comparison to the soluble trans-IPPS proteins. In addition to sharing a fold with the UbiA homologs, the two protein families also exhibit similarities in the architecture of their active sites. The structure of *E. coli* FPPS bound to IPP and a thio- analog of the prenyl donor, thioDMAPP, is representative of available structures of trans-IPPS ternary complexes (Figure S9) [21]. Like AfUbiA, members of the trans-IPPS family contain two signature acidic motifs, which both contain the conserved sequence DDXXD [36] and which coordinate Mg^2+^ atoms that stabilize the diphosphate on the prenyl donor. In trans-IPPS proteins, the reaction is believed to proceed via a three-stage ionization–condensation–elimination mechanism [37], involving a carbocation intermediate in the allylic site that is stabilized by the liberated diphosphate as well as interactions with nearby polar side chains [21],[38]. Given the structural and functional similarity between AfUbiA and the trans-IPPS proteins, it is possible that this catalytic mechanism is shared with homologs of the UbiA family (Figure 6).

In the GPP- and DMAPP-bound structures, C-C bond formation would occur near a triad of three polar side chains: N68 from the first aspartate-rich motif, and Y139 and S140 on TM4 (Figure 3B). Interestingly, the tyrosine is the single most highly conserved residue in the UbiA family, present in 97% of the more than 10,000 UbiA sequences currently in the Pfam database [39]. Mutation of this residue in both EcMenA (Figure 5C,D) and EcUbiA [19] results in a loss of function. The near-universal conservation of the tyrosine residue implies that this site is involved in a function that is shared among all the different branches of the UbiA family, regardless of the nature of the highly variable prenyl acceptor. We therefore propose that this site could be involved in stabilizing the carbocation intermediate on the prenyl donor after cleavage of the pyrophosphate leaving group, possibly by cation–π interactions with Y139. A similar role for active site tyrosine residues has been proposed for the structurally unrelated aromatic prenyltransferases DMATS and CloQ [40]–[42].

## Discussion

We have described the structure of an archaeal member of the UbiA family of membrane-embedded prenyltransferases. The substrate-bound structures reveal that the diphosphate group interacts with arginine and lysine side chains as well as Mg^2+^ coordinated by conserved aspartate-rich motifs. Although short prenyl donors containing only one or two prenyl units were chosen for crystallization due to their higher solubility in water, the structure reveals a long, narrow cavity that opens into the membrane and could allow the protein to bind significantly longer polyprenyl chains. Due in part to its low homology with UbiA family members of known function, we were unable to identify the natural prenyl-accepting substrate of AfUbiA. Nonetheless, the EcMenA functional assays using mutations designed with the AfUbiA structure, as well as the clustering of SCD-causing mutations around conserved residues in the substrate-binding cavity of AfUbiA, suggest that the fold and key aspects of the catalytic mechanism may be conserved between these distantly related homologs and that AfUbiA is a useful structural model for understanding UbiA family prenyltransferases.

One curious feature of the substrate-bound structures is that the Mg^2+^ bound to the second conserved motif is out of range for coordination of the prenyl donor, and yet the functional data for EcMenA indicate that the residues coordinating this Mg^2+^ are critical for function. One possible explanation is that when the protein is bound to Mg^2+^ and the isoprenyl diphosphate only, the substrate is coordinated by only one Mg^2+^ in order to prevent reaction of the isoprenyl diphosphate with water in the absence of the prenyl acceptor, as interactions with one Mg^2+^ may not be sufficient to induce spontaneous cleavage of the C–O bond. Binding of the prenyl acceptor would then induce a conformational change to bring the Mg^2+^ bound to the second conserved motif within coordination distance of the diphosphate and exclude water from the cavity. Another possibility has also been proposed based on homology models of *E. coli* UbiA [23], that UbiA homologs stabilize the diphosphate via a single Mg^2+^ bound to only one of the two aspartate-rich motifs and that the other motif activates the phenolic substrate by abstracting a proton. Resolution of the issue will likely require a structure of the full ternary complex.

In the structurally related trans-IPPS FPPS, comparisons of the ternary complex and apo-structure show that the enzyme undergoes a conformational change upon binding substrate; in particular, the loops corresponding to L2–3 and L6–7 in UbiA close over the active site to occlude it from the solvent [21]. The unliganded and substrate-bound AfUbiA structures show similar behavior, in that a region of the L2–3 loop undergoes a disordered to ordered transition upon substrate binding. The prenyl acceptor could therefore enter the cavity through an opening formed by fluctuations in the L2–3 loop, which would then be stabilized in the closed conformation. Although short polyprenyl diphosphates like GPP could also enter the substrate-binding cavity via the opening to the cytoplasm, longer polyprenyl diphosphates have poor solubility in water and likely partition into the lipid bilayer, and therefore may bind to the protein laterally from within the membrane. Because it seems implausible that the negatively charged diphosphate group enters the core of the bilayer and threads into the hydrophobic tunnel, the protein may undergo a conformational change to allow the polyprenyl substrate access to the substrate binding cavity. In the crystal structure, one wall of the substrate tunnel is formed by TM9, whose removal leaves the central cavity completely exposed to the bilayer (Figure 2A). A slight movement of this loosely packed helix could potentially suffice to allow substrates to enter the binding site and allow release of the product as well.

## Methods

### Cloning and Overexpression of AfUbiA

Fifty-two bacterial and archaeal UbiA homologs were cloned and tested for expression [43]. A UbiA gene from *Archaeoglobus fulgidus* DSM 4304 (GenBank AAB89594.1) was identified as the most promising candidate for crystallization trials. The AfUbiA gene was cloned into a modified pET vector (Novagen) with an N-terminal polyhistidine tag. For large-scale purification of native AfUbiA, the plasmid was transformed into BL21 (DE3) cells. For expression of the native protein, the transformants were grown in Luria broth supplemented with 100 mg/l Kanamycin at 37°C until OD_600_ reached 1.0 and induced with 0.5 mM isopropyl β-D-1-thiogalactopyranoside (IPTG) at 20°C for 15 h. For expression of selenomethionine-incorporated proteins, the cells were grown in minimal medium containing 32.2 mM K_2_HPO_4_, 11.7 mM KH_2_PO_4_, 6 mM (NH_4_)_2_SO_4_, 0.68 mM Na Citrate, 0.17 mM Mg_2_SO_4_, 32 mM glucose, 0.008% (w/v) alanine, arginine, aspartic acid, asparagine, cysteine, glutamic acid, glycine, histidine, proline, serine, tryptophan, glutamine, tyrosine, 0.02% (w/v) isoleucine, leucine, lysine, phenylalanine, threonine, valine, 25 mg/l L-selenium-methionine, 32 mg/l thiamine, and 32 mg/l thymine, and induced when OD_600_ reached 0.6.

### Purification and Crystallization of AfUbiA

Cell membranes were solubilized with 40 mM n-decyl-β-D-maltoside (DM, Anatrace), and the His-tagged protein was purified with TALON Metal Affinity Resin (Clontech Inc.). After removal of the N-terminal His-tag with TEV protease, the native protein was subjected to size exclusion chromatography with a Superdex 200 10/300 GL column (GE Health Sciences) pre-equilibrated in a buffer of 150 mM NaCl, 20 mM HEPES, pH 7.5, 5 mM β-mercaptoethanol (βME), and 40 mM n-Octyl-β-D-Glucopyranoside (OG, Affymetrix). The protein was concentrated to 10 mg/ml as approximated by ultraviolet absorbance. The selenomethionine-incorporated protein was purified by the same procedure. AfUbiA for the ITC assay was also purified with the same protocol except that 4 mM of DM was used in place of OG in the size-exclusion chromatography buffer.

Selenomethionine-incorporated AfUbiA crystals were obtained in mother liquor containing 12.5% PEG20000, 100 mM MES buffer, pH 6.7. To obtain LCP crystals, the purified AfUbiA protein was concentrated to around 35 mg/ml as approximated by ultraviolet absorbance at 280 nm and mixed with monoolein (1-oleoyl-rac-glycerol; Sigma Aldrich) at a 2∶3 ratio (protein/lipid, w/w) using the twin-syringe mixing method [44]. The protein/lipid mixture was dispensed manually in 30–50 nl drops onto 96-well glass Laminex plates (Molecular Dimensions) and overlaid with 1.7 µl precipitant solution per drop. Crystals reached full size within 2 wk at 20°C in 34% (w/v) PEG400, 0.1 M Tris-HCl pH 8.2, 0.1 M NaCl, and 0.1 M MgCl_2_. Before harvest, crystals were soaked in 1 mM GPP or 1 mM DMAPP. The LCP crystals were flash frozen in liquid nitrogen without additional cryoprotectant. Crystals of native AfUbiA bound to Cd^2+^ were obtained in 30% PEG 550 MME, 100 mM MES buffer, pH 6.6, 5 mM MgCl_2_, and 100 mM CdCl_2_. Before flash-freezing in liquid nitrogen, these crystals were cryoprotected in serial mother liquor solutions containing 5%–25% (v/v) glycerol.

### Structure Solution and Analysis

X-ray data were collected at beamlines X29 at the National Synchrotron Light Source and 24ID-C and 24ID-E at the Advanced Photon Source. A data set collected on a selenomethionine crystal was processed and scaled with a 3.2 Å cutoff using HKL2000 [45]. Four selenium sites were located with phenix.hyss [46], and phases and a partial polyalanine model were obtained with phenix.autosol [47]. The locations of the selenium atoms and clear side chain densities from aromatic side chains in the experimental maps (Figure S2B,C) were used to manually assign the sequence register, and the structure was refined through iterative rounds of manual model building and automated reciprocal-space refinement using Coot [48] and phenix.refine. The final refined model has R and R_free_ values of 25.1% and 28.9%, respectively, and contains residues 15–73 and 86–300 of one UbiA monomer and one molecule of OG, which was used to solubilize the protein. The native GPP-bound and DMAPP-bound structures were solved by molecular replacement using the selenomethionine structure as a search model and refined with phenix.refine using strong NCS restraints that were gradually relaxed over the course of refinement. The final structures each contained four molecules of AfUbiA, 8 Mg^2+^, and 4 molecules of GPP or DMAPP. The Cd^2+^-bound structure was solved by a similar protocol. The final Cd^2+^-bound structure contained two molecules of AfUbiA in the asymmetric unit and eight Cd^2+^ ions. In the SeMet, GPP- and DMAPP-bound structures, the putative substrate tunnel is partly occupied by a strong, tubular, nonprotein electron density (); however, the resolutions do not allow a definitive identification of this ligand. The GPP-bound, DMAPP-bound, SeMet, and Cd^2+^-bound structures have been deposited in the PDB under the accession codes 4TQ3, 4TQ4, 4TQ5, and 4TQ6, respectively.

Chain A in the GPP-bound structure had the highest quality 2F_o_-F_c_ density, as well as the lowest average B-factors of the four protein chains in the asymmetric unit, and was therefore used to generate figures and for distance measurements unless otherwise noted. All structure figures were made using PyMol (Schrödinger). Sequence conservation scores in Figure 2B were calculated with the ConSurf server [49], using the seed sequences for the UbiA family from Pfam [39] for the multiple sequence alignment. The alignment of AfUbiA, EcMenA, and human UBIAD1 used for Figure 5A was generated by aligning the sequences to the Hidden Markov Model profile for the UbiA family in Pfam.

### ITC of GPP Binding to AfUbiA

The ITC buffer comprised 20 mM HEPES (pH 7.5), 150 mM NaCl, and 4 mM n-decyl-β-D-maltopyranoside (DM). The chamber contained ITC buffer plus 50 µM AfUbiA and either 2 mM MgCl_2_ or 1 mM EDTA. The syringe contained ITC buffer plus 0.6 mM geranyl pyrophosphate (GPP) and either 2 mM MgCl_2_ or 1 mM EDTA, whichever matches the chamber condition. The buffer-alone control had no AfUbiA in the chamber. For experiments with mutant proteins, 50 µM AfUbiA mutant proteins were in ITC buffer containing 2 mM MgCl_2_ with either 0.6 mM GPP (for R22A, N68A, D72A, K146A) or 2 mM GPP (for D198A and D202A) in the syringe. Solutions were filtered and centrifuged at 18,000× *g* for 5 min prior to the experiments. All binding measurements were performed using a MicroCal iTC200 System (GE Healthcare) at a constant temperature of 25°C. For experiments with apparent binding, thermograms were processed and fit in Origin to a one-site model to obtain n (stoichiometry), K (association constant), and ΔH (enthalpy). The dissociation constant (K_D_) was calculated from K_D_ = 1/K, and ΔS was calculated from ΔG = ΔH−TΔS. All experiments were performed at least three times.

### *MenA* Complementation Assay

The *menA*-deficient *E. coli* strain AN67 [25], which exhibits a grow defect under anaerobic conditions, was obtained from the Coli Genetic Stock Center and transformed with a pET31 plasmid containing WT and mutant EcMenA genes, or an unrelated protein (the TrkH potassium transporter from *Campylobacter jejuni*) as a negative control. The transformants were grown aerobically in Luria broth to an optical density of 1.0%±0.05%, supplemented with 20% glycerol, flash frozen, and stored at −80°C. The EcMenA genetic rescue experiments were carried out using a protocol adapted from Suvarna et al. [7]. The 10 mL cultures of a glycerol/trimethylamine N-oxide minimal media [50] containing 0.5 mM IPTG and 0.1 mg/mL ampicillin were inoculated with 10 µl of the glycerol stocks and then incubated in an anaerobic chamber at 37°C. At 24 h postinoculation, OD_600_ was measured for each culture. Values shown in Figure 5C are averages for three experiments.

### Prenyltransferase Activity Assay

Purified *E. coli* membranes were prepared by harvesting 1 l of cells overexpressing WT and mutant EcMenA, grown to 1 OD as described for AfUbiA. The proteins were expressed as SUMO fusion proteins to increase yield. The cell pellets were resuspended in 20 ml lysis buffer (20 mM Hepes pH 7.5, 150 mM NaCl, 2 mM βME, 5 mM MgCl_2_, 1 mM PMSF, 25 µg/ml DnaseI). After breaking the cells by sonication, the cell lysates were centrifuged for 30 min at 3,000× *g* and 4°C. The supernatant was then transferred to clean centrifuge tubes and centrifuged a second time for 60 min at 100,000× *g* and 4°C. The membrane pellet was resuspended in 3 ml 50 mM Tris pH 7.5, flash frozen, and stored at −80°C. Overexpression of WT and mutant SUMO-EcMenA was verified by running 0.5 µl of the membrane suspension before and after a 30 min digestion with 1 µg SUMO protease on an SDS-PAGE gel (Figure S8C). In addition to the WT and mutant EcMenA proteins, membrane fractions were prepared in the same manner for cells expressing SUMO-EcUbiA, which does not utilize DHNA as a prenyl acceptor, for the negative control.

For the enzymatic assay, 30 µl reaction mixtures were prepared with the following components: 3 µl purified membrane fractions, 2 mM DHNA, 1 mM GPP, 5 mM MgCl_2_, 5 mM βME, 5% acetonitrile (ACN), and 50 mM Tris pH 7.5. The reaction mixtures were incubated for 10 min at 37°C, quenched with the addition of 2% formic acid, and extracted with 10 volumes of chloroform. The chloroform was dried under air and the residue resuspended in 60 µl 65% ACN/35% 50 mM Tris pH 7.5 in dH_2_O. The resulting samples were then separated using reverse phase HPLC with a gradient of 65%–75% ACN for the mobile phase. Enzyme activity was quantified as the area of the product peak, normalized by the activity for the WT protein. Values shown in Figure 5D are averages for three experiments.

## Supporting Information

Figure S1 Metabolic pathway for the conversion of phylloquinone to menaquinone-4. FPPS, farnesyl diphosphate synthase; GGPPS, geranylgeranyl diphosphate synthase. The enzyme responsible for cleaving the phytyl tail from phylloquinone is not currently known.
(TIF)

Figure S2 Purification and structure solution of *Archaeoglobus fulgidus* UbiA. (A) Elution profile of AfUbiA solubilized in the detergent β-octylglucoside from a size-exclusion column. (B) The structure of AfUbiA overlaid with electron density from the anomalous difference map contoured at 4 σ (orange mesh). Selenium atoms from selenomethionine residues are shown as blue spheres. (C) A representative region of the electron density map calculated from the experimental phases, after solvent flattening and density modification, for the SeMet/detergent crystal used for phasing. The blue mesh corresponds to a contour level of 1.5 σ. (D) The same region in the 2F_o_-F_c_ maps in the GPP-bound native structure, contoured at 1.5 σ. (E) One cross-section of the crystal lattice in the P3_1_12 detergent crystals (left) and the P2_1_ LCP crystals (right). Molecules from one asymmetric unit in each are colored red.
(TIF)

Figure S3 Pseudosymmetry in the UbiA fold. (A) Transmembrane helices TM1–4 (left) and TM5–8 (right) of AfUbiA. (B) TM1–4 and TM5–8 are shown superposed on each other from two different orientations.
(TIF)

Figure S4 Structural similarity to soluble isoprenoid synthases. (A–B) The structures of AfUbiA (A) and an FPPS from *E. coli* (PDB accession code 1RQI) (B) are shown as cartoon representations from the same orientation. For consistency with AfUbiA, the helices in 1RQI are numbered 0–8 and the helices in both proteins are colored according to the same scheme as in Figure 1C. (C–D) The structures of AfUbiA (C) and 1RQI (D) are shown as cartoon representations from the same orientation. All histidine, lysine, and arginine residues in both structures are shown as blue spheres, and all aspartate and glutamate residues are shown as red spheres.
(TIF)

Figure S5 The DMAPP-bound structure of AfUbiA. (A) A ribbon representation of the DMAPP-bound AfUbiA structure in which the thickness of the ribbon indicates the magnitude of the temperature factor. Residues that are resolved in the DMAPP-bound structure but disordered in the unliganded structure are highlighted in red. (B) A cutaway surface of the DMAPP-bound structure, showing that the central cavity is occluded from the solvent. (C–D) Stereoviews of the active site in the DMAPP-bound structure from two orientations. Green mesh corresponds to the F_o_-F_c_ map calculated with ligand and water molecules omitted, contoured at 3.0 σ.
(TIF)

Figure S6 Ion binding sites in the central cavity. (A) Stereo view of the active site in the Cd^2+^-bound structure, viewed from the cytoplasmic side of the membrane. Yellow spheres correspond to two Cd^2+^ atoms, and purple spheres correspond to the locations of Mg^2+^ atoms in the GPP-bound structure when superposed with the Cd^2+^ structure. Residues that bind to Mg^2+^ and the diphosphate are labeled. (B) Stereo view of the active site from within the plane of the membrane. Conserved residues predicted to stabilize the intermediate state are labeled. The green mesh in both figures corresponds to F_o_-F_c_ density contoured at 4.0 σ.
(TIF)

Figure S7 GPP binding to AfUbiA mutant proteins measured by ITC. (A) Thermograms of four mutant proteins with no detected GPP binding. (B) Thermograms (top) for two mutant proteins with measurable affinities for GPP and their corresponding binding isotherms (bottom). (C) Thermogram of 2 mM GPP injected into the ITC chamber with no protein present.
(TIF)

Figure S8 EcMenA prenyltransferase assay. (A–B) Membranes were purified from *E. coli* overexpressing SUMO-EcMenA or SUMO-EcUbiA and incubated at 37°C for 10 min with 2 mM DHNA, 1 mM GPP, and 5 mM MgCl_2_. The reaction mixtures were then extracted with chloroform and separated by reverse phase HPLC. Representative HPLC traces are shown for (A) WT EcMenA and (B) WT EcUbiA used as a negative control. The product peak is marked with an arrow. (C) To verify that all SUMO-EcMenA mutants were overexpressed, 0.5 µl of purified membrane was run on an SDS-PAGE gel. Lanes marked with minus and plus signs indicate whether samples were cleaved with SUMO protease prior to loading on the gel.
(TIF)

Figure S9 The soluble polyprenyl synthase fold. (A–B) The structure of a FPPS from *E. coli* (PDB accession code 1RQI) is shown from two perpendicular orientations. For consistency with AfUbiA, the helices are numbered 0–8 and colored according to the same scheme as in Figure 1C. Orange arrows indicate the two pseudosymmetric bundles. (C) Two perpendicular views of the binding pocket of FPPS bound to Mg^2+^, thioDMAPP, and IPP. In the left panel, the red asterisk marks the bond that is cleaved in DMAPP, and the red arrow indicates where a new bond is formed between IPP and DMAPP.
(TIF)

Figure S10 Electron density in the putative substrate tunnel. (A) Electron density in the putative substrate channel of AfUbiA in the experimental maps from the SeMet, unliganded dataset, contoured at 1.5 σ. (B) F_o_-F_c_ density contoured at 3.0 σ in the same region calculated from the dataset for the GPP-bound structure.
(TIF)

Table S1 Data collection and refinement statistics.
(DOC)

Table S2 Complementation of *menA^−^ E. coli* by WT and mutant EcMenA. OD_600_ measurements from three experiments of *E. coli* cultures grown under anaerobic conditions, used to calculate the bar graph in Figure 5C.
(DOC)

Table S3 Prenyltransferase activities of WT and mutant EcMenA. Normalized activities from three experiments with WT and mutant EcMenA, used to calculate the bar graph in Figure 5D.
(DOC)

## Abbreviations

4HB: 4-hydroxybenzoate
DHNA: 1,4-dihydroxy-2-naphthoic acid
DMAPP: dimethylallyl diphosphate
FPPS: farnesyl diphosphate synthase
GPP: geranyl diphosphate
IPP: isopentenyl pyrophosphate
IPPS: isoprenyl diphosphate synthase
ITC: isothermal titration calorimetry
LCP: lipidic cubic phase
SCD: Schnyder corneal dystrophy
UBIAD1: UbiA prenyltransferase domain-containing 1
