# Supplementary figures and images for: Structure of a Membrane-Embedded Prenyltransferase Homologous to UBIAD1
Source: PLoS Biol. 2014 Jul 22;12(7):e1001911. doi: 10.1371/journal.pbio.1001911 (PMC4106721; doi:10.1371/journal.pbio.1001911)

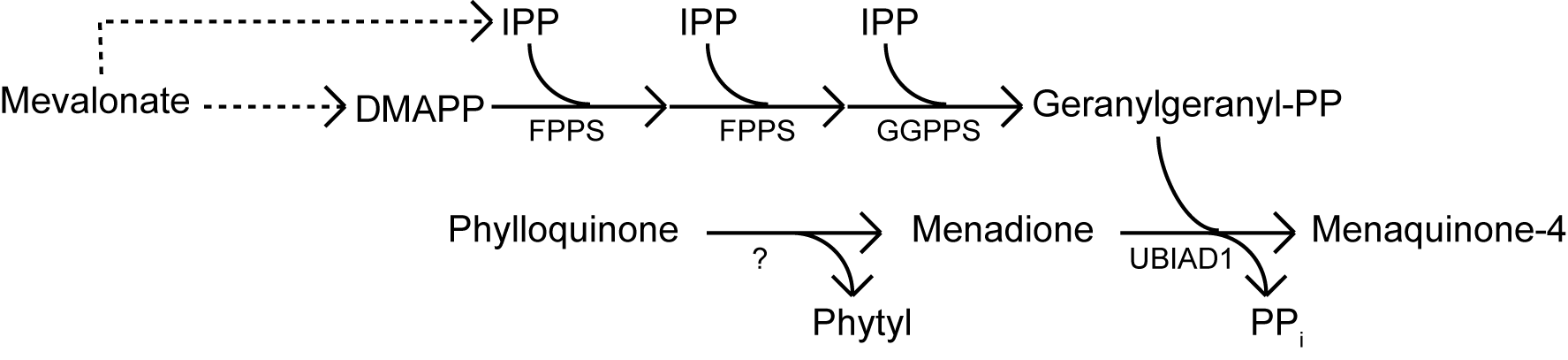

Supplement: Figure S1 — Metabolic pathway for the conversion of phylloquinone to menaquinone-4. FPPS, farnesyl diphosphate synthase; GGPPS, geranylgeranyl diphosphate synthase. The enzyme responsible for cleaving the phytyl tail from phylloquinone is not currently known. (TIF) [file pbio.1001911.s001.tif]

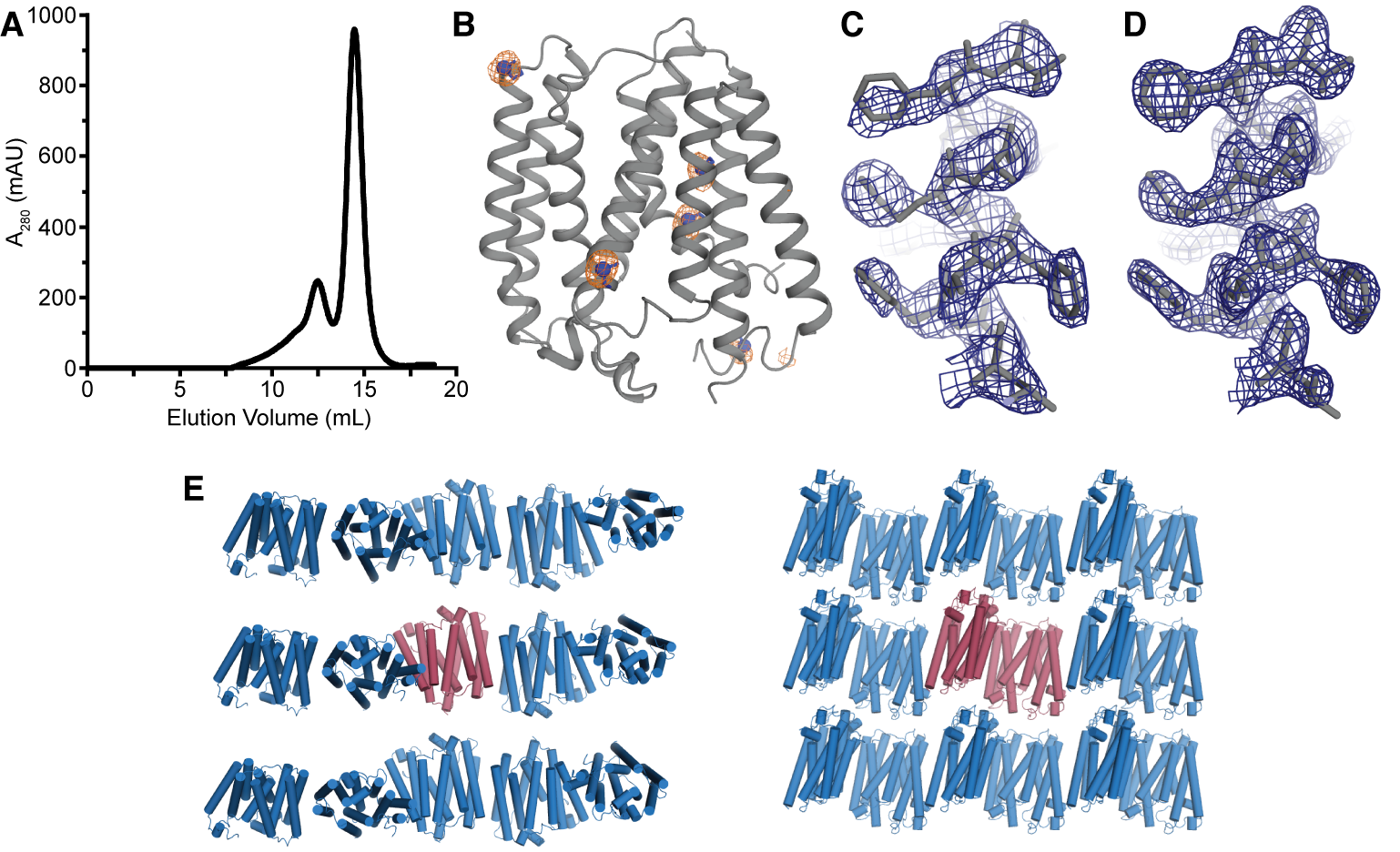

Supplement: Figure S2 — Purification and structure solution of Archaeoglobus fulgidus UbiA. (A) Elution profile of AfUbiA solubilized in the detergent β-octylglucoside from a size-exclusion column. (B) The structure of AfUbiA overlaid with electron density from the anomalous difference map contoured at 4 σ (orange mesh). Selenium atoms from selenomethionine residues are shown as blue spheres. (C) A representative region of the electron density map calculated from the experimental phases, after solvent flattening and density modification, for the SeMet/detergent crystal used for phasing. The blue mesh corresponds to a contour level of 1.5 σ. (D) The same region in the 2Fo-Fc maps in the GPP-bound native structure, contoured at 1.5 σ. (E) One cross-section of the crystal lattice in the P3112 detergent crystals (left) and the P21 LCP crystals (right). Molecules from one asymmetric unit in each are colored red. (TIF) [file pbio.1001911.s002.tif]

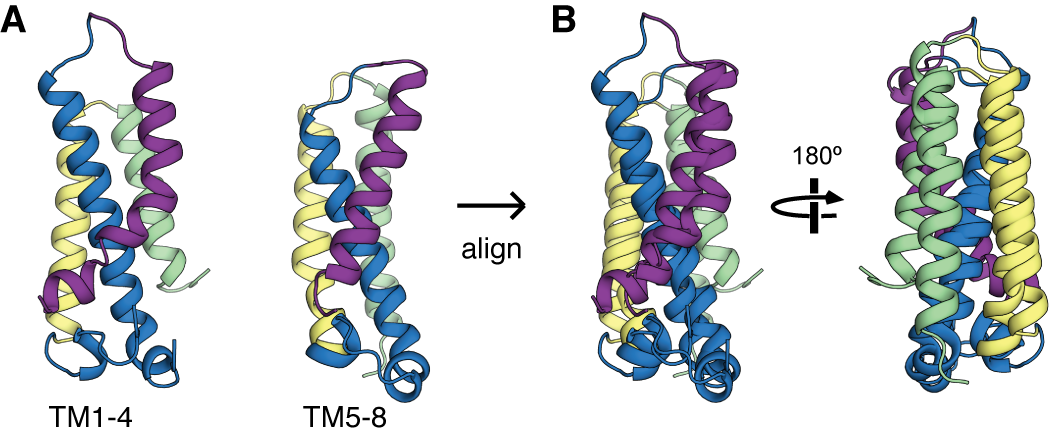

Supplement: Figure S3 — Pseudosymmetry in the UbiA fold. (A) Transmembrane helices TM1–4 (left) and TM5–8 (right) of AfUbiA. (B) TM1–4 and TM5–8 are shown superposed on each other from two different orientations. (TIF) [file pbio.1001911.s003.tif]

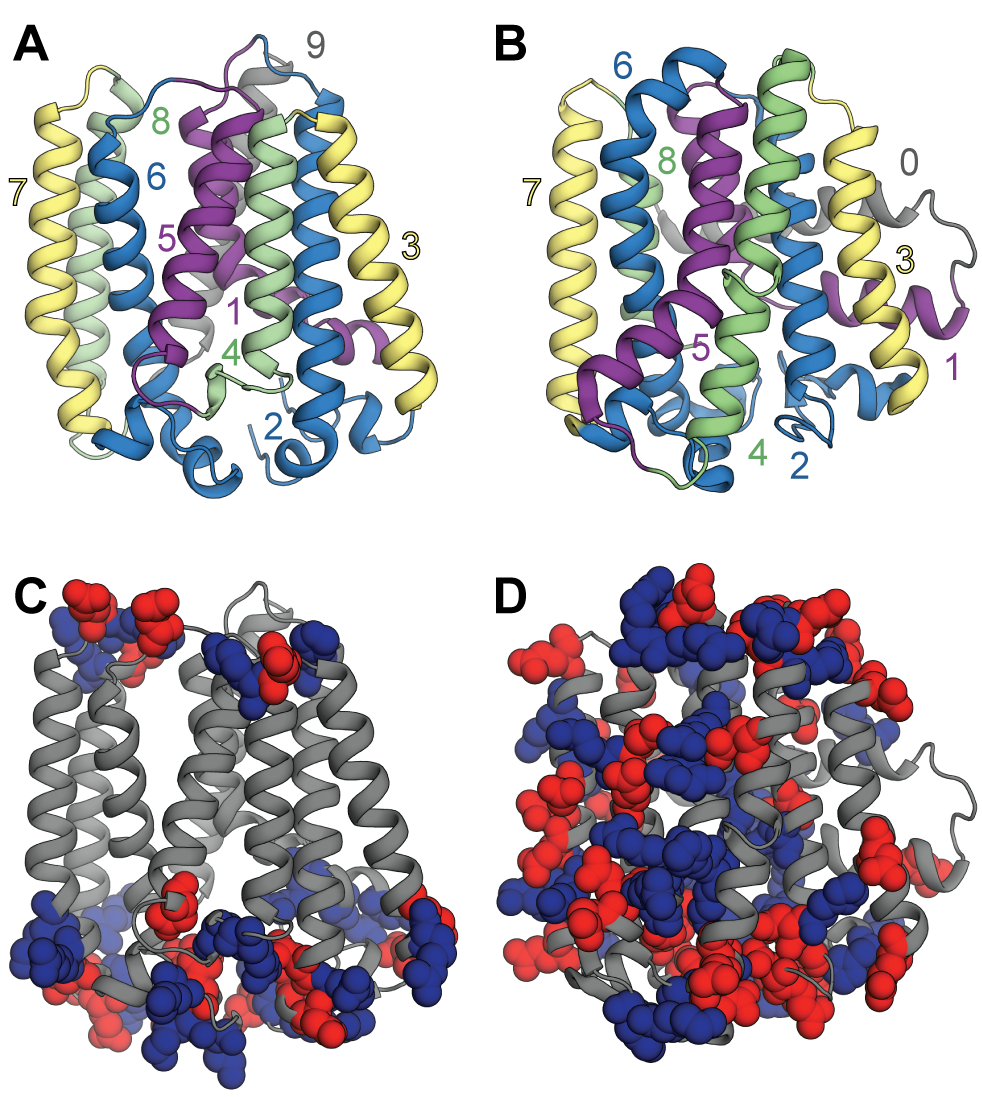

Supplement: Figure S4 — Structural similarity to soluble isoprenoid synthases. (A–B) The structures of AfUbiA (A) and an FPPS from E. coli (PDB accession code 1RQI) (B) are shown as cartoon representations from the same orientation. For consistency with AfUbiA, the helices in 1RQI are numbered 0–8 and the helices in both proteins are colored according to the same scheme as in Figure 1C. (C–D) The structures of AfUbiA (C) and 1RQI (D) are shown as cartoon representations from the same orientation. All histidine, lysine, and arginine residues in both structures are shown as blue spheres, and all aspartate and glutamate residues are shown as red spheres. (TIF) [file pbio.1001911.s004.tif]

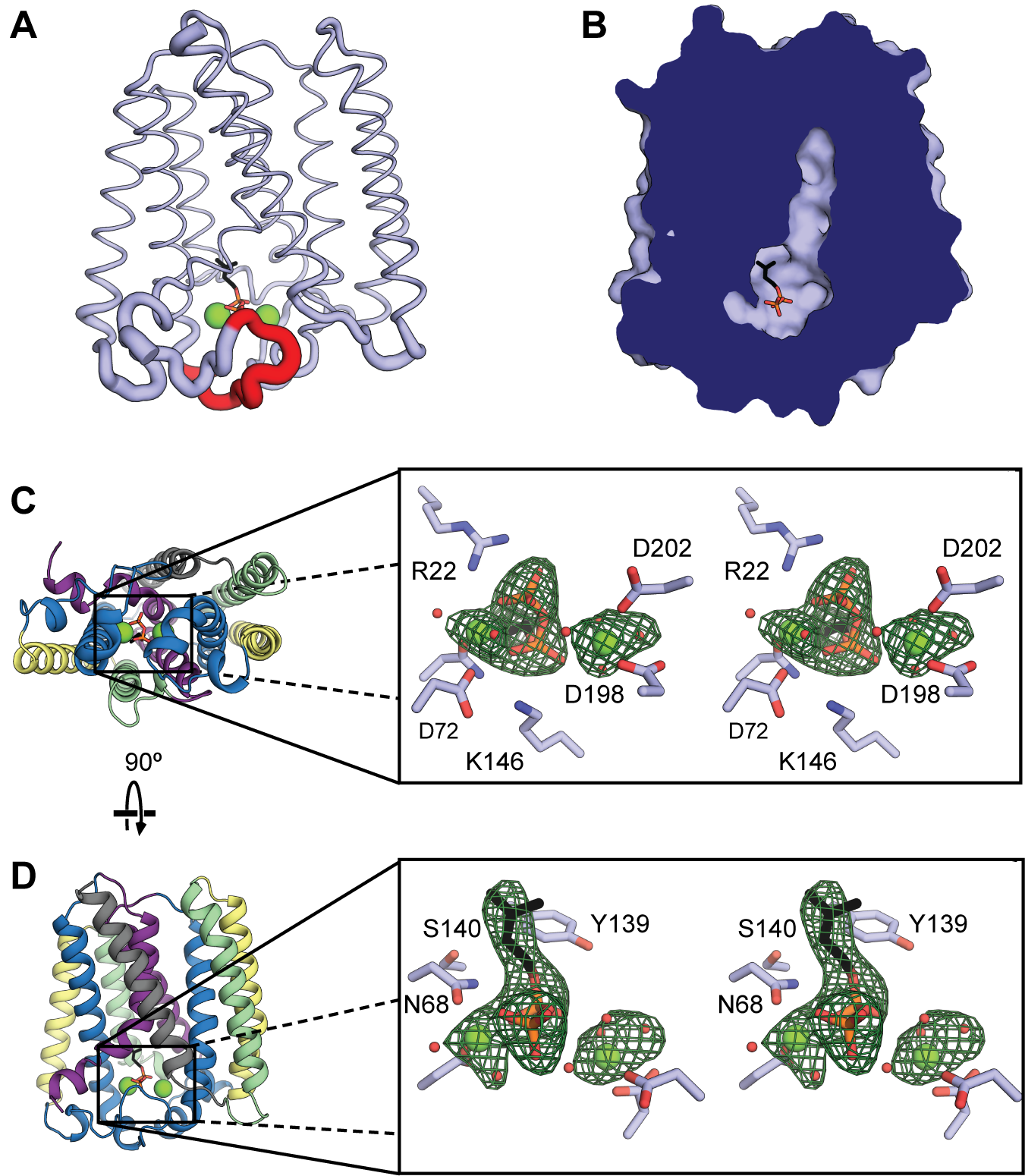

Supplement: Figure S5 — The DMAPP-bound structure of AfUbiA. (A) A ribbon representation of the DMAPP-bound AfUbiA structure in which the thickness of the ribbon indicates the magnitude of the temperature factor. Residues that are resolved in the DMAPP-bound structure but disordered in the unliganded structure are highlighted in red. (B) A cutaway surface of the DMAPP-bound structure, showing that the central cavity is occluded from the solvent. (C–D) Stereoviews of the active site in the DMAPP-bound structure from two orientations. Green mesh corresponds to the Fo-Fc map calculated with ligand and water molecules omitted, contoured at 3.0 σ. (TIF) [file pbio.1001911.s005.tif]

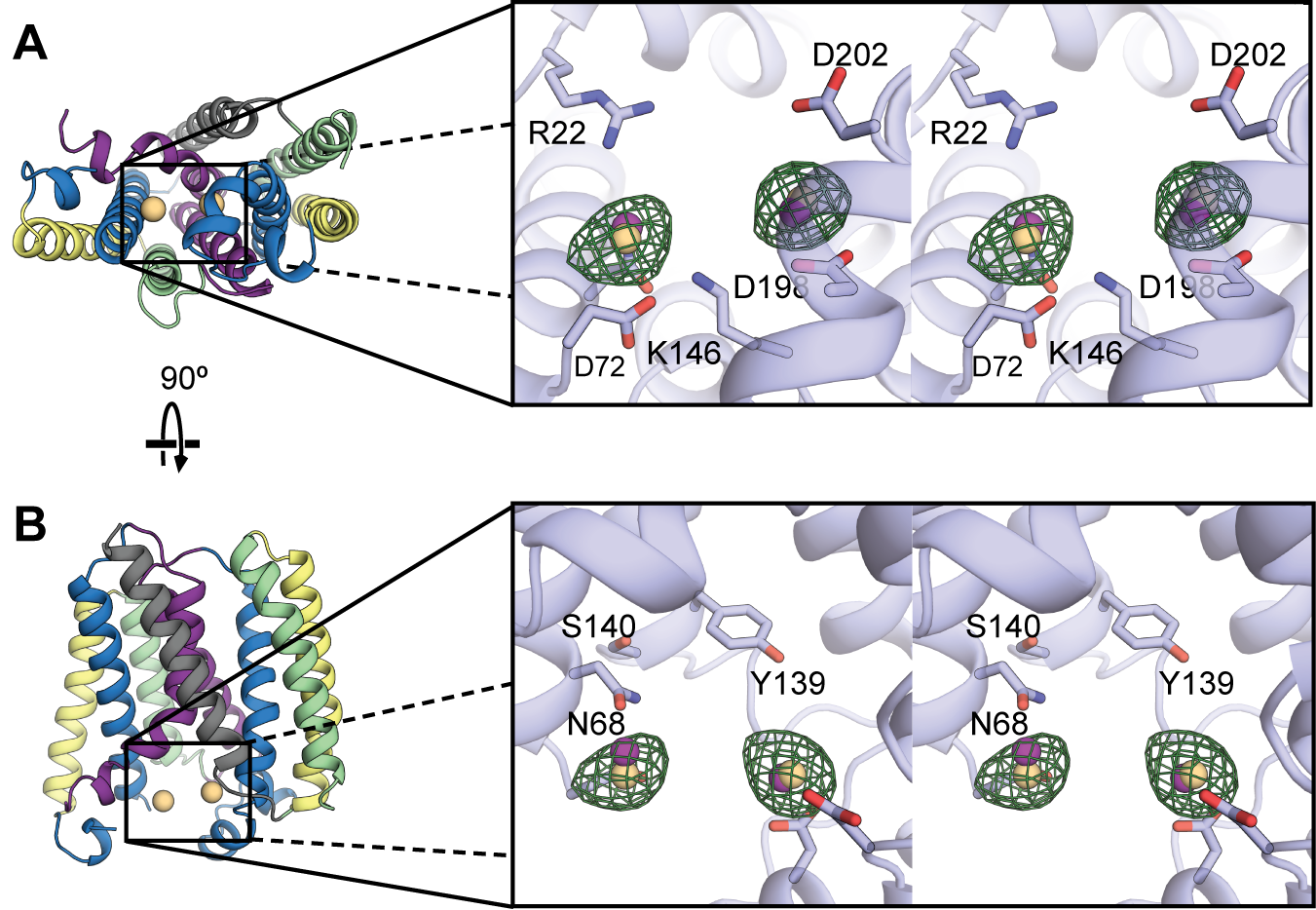

Supplement: Figure S6 — Ion binding sites in the central cavity. (A) Stereo view of the active site in the Cd2+-bound structure, viewed from the cytoplasmic side of the membrane. Yellow spheres correspond to two Cd2+ atoms, and purple spheres correspond to the locations of Mg2+ atoms in the GPP-bound structure when superposed with the Cd2+ structure. Residues that bind to Mg2+ and the diphosphate are labeled. (B) Stereo view of the active site from within the plane of the membrane. Conserved residues predicted to stabilize the intermediate state are labeled. The green mesh in both figures corresponds to Fo-Fc density contoured at 4.0 σ. (TIF) [file pbio.1001911.s006.tif]

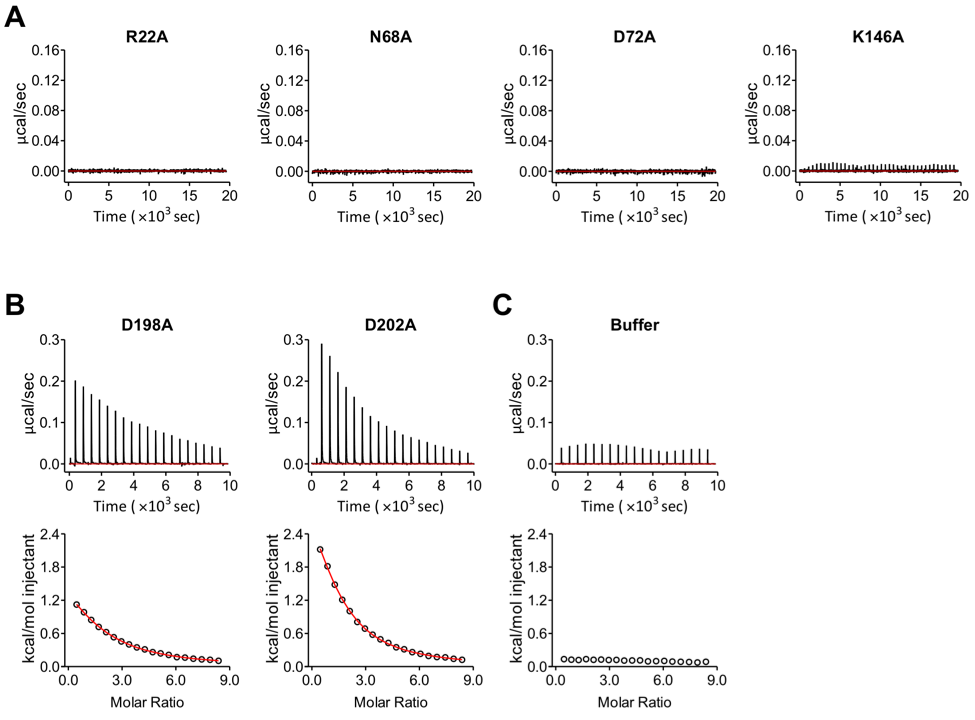

Supplement: Figure S7 — GPP binding to AfUbiA mutant proteins measured by ITC. (A) Thermograms of four mutant proteins with no detected GPP binding. (B) Thermograms (top) for two mutant proteins with measurable affinities for GPP and their corresponding binding isotherms (bottom). (C) Thermogram of 2 mM GPP injected into the ITC chamber with no protein present. (TIF) [file pbio.1001911.s007.tif]

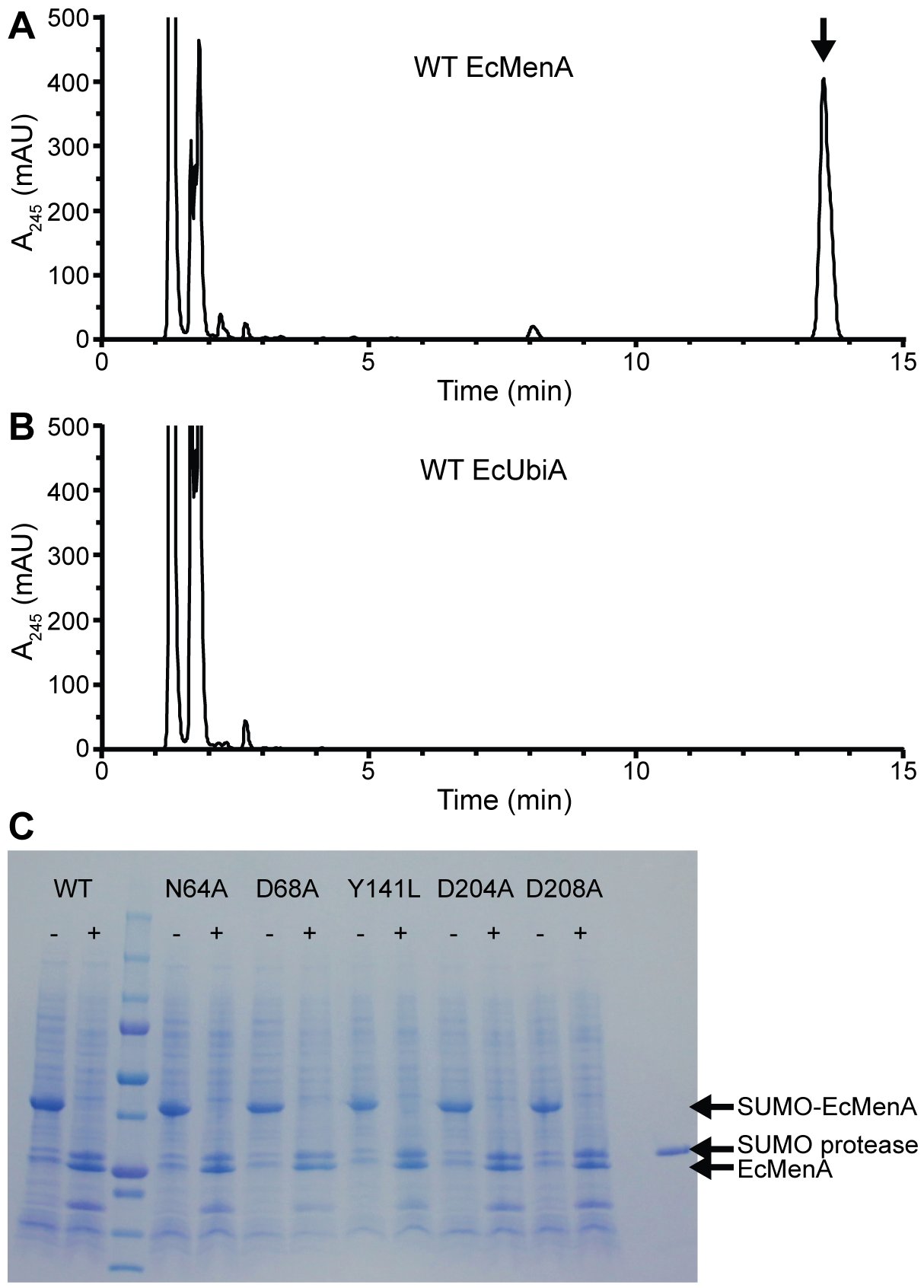

Supplement: Figure S8 — EcMenA prenyltransferase assay. (A–B) Membranes were purified from E. coli overexpressing SUMO-EcMenA or SUMO-EcUbiA and incubated at 37°C for 10 min with 2 mM DHNA, 1 mM GPP, and 5 mM MgCl2. The reaction mixtures were then extracted with chloroform and separated by reverse phase HPLC. Representative HPLC traces are shown for (A) WT EcMenA and (B) WT EcUbiA used as a negative control. The product peak is marked with an arrow. (C) To verify that all SUMO-EcMenA mutants were overexpressed, 0.5 µl of purified membrane was run on an SDS-PAGE gel. Lanes marked with minus and plus signs indicate whether samples were cleaved with SUMO protease prior to loading on the gel. (TIF) [file pbio.1001911.s008.tif]

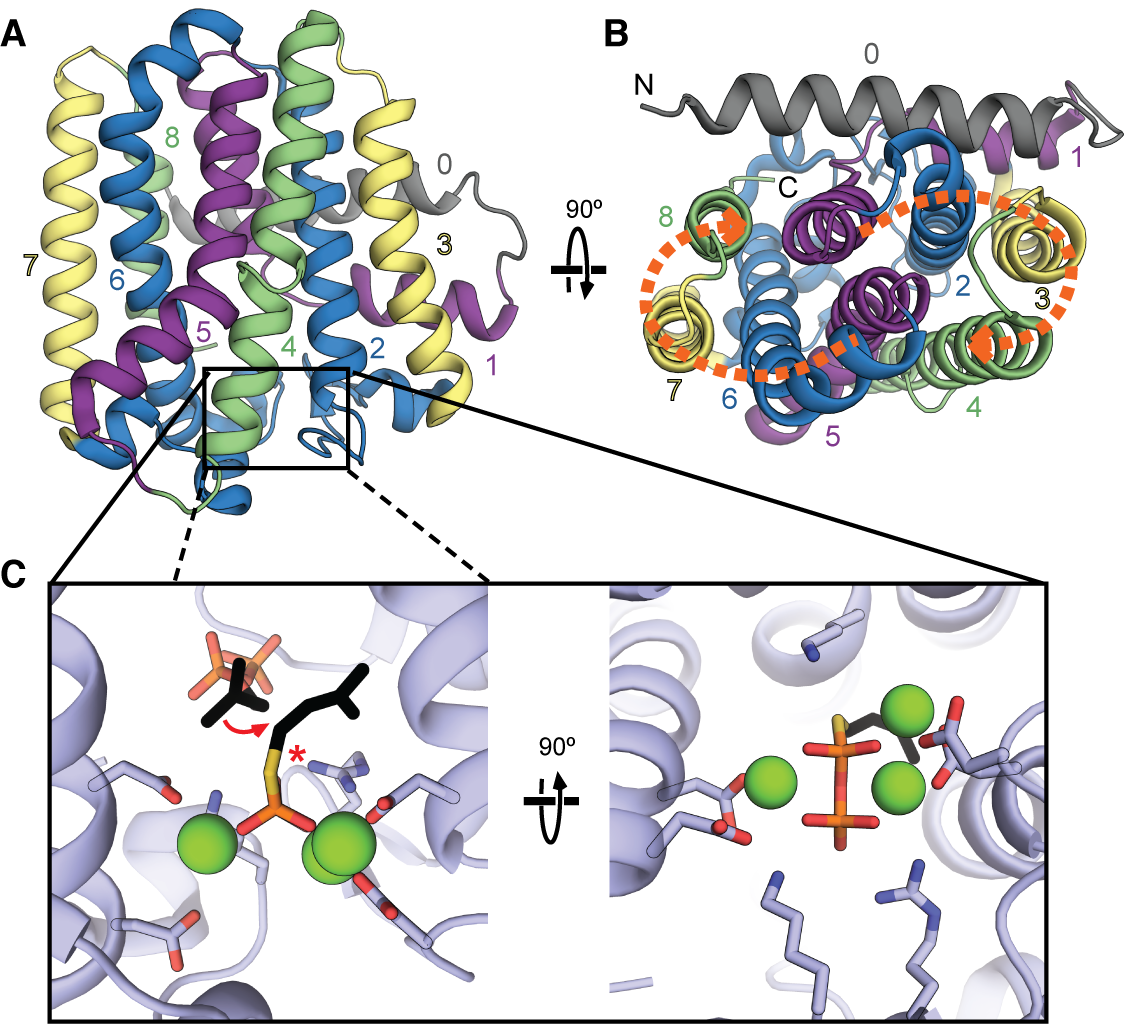

Supplement: Figure S9 — The soluble polyprenyl synthase fold. (A–B) The structure of a FPPS from E. coli (PDB accession code 1RQI) is shown from two perpendicular orientations. For consistency with AfUbiA, the helices are numbered 0–8 and colored according to the same scheme as in Figure 1C. Orange arrows indicate the two pseudosymmetric bundles. (C) Two perpendicular views of the binding pocket of FPPS bound to Mg2+, thioDMAPP, and IPP. In the left panel, the red asterisk marks the bond that is cleaved in DMAPP, and the red arrow indicates where a new bond is formed between IPP and DMAPP. (TIF) [file pbio.1001911.s009.tif]

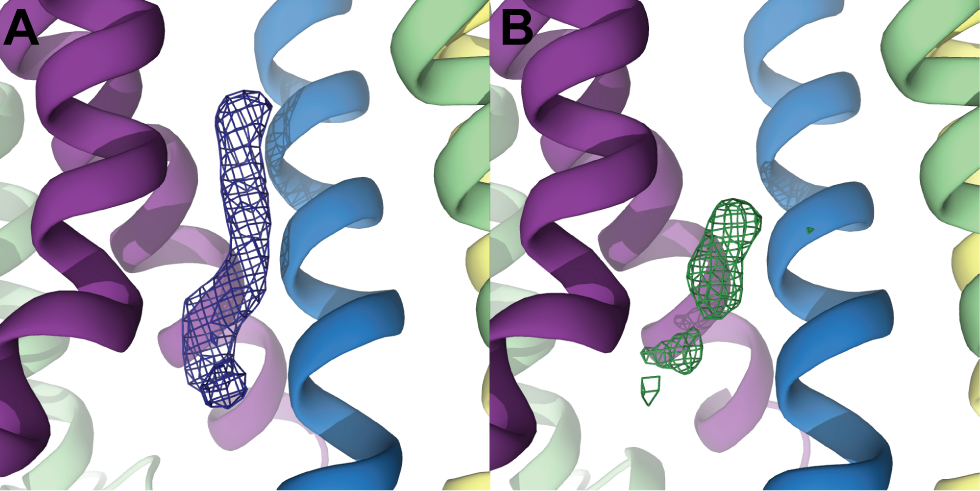

Supplement: Figure S10 — Electron density in the putative substrate tunnel. (A) Electron density in the putative substrate channel of AfUbiA in the experimental maps from the SeMet, unliganded dataset, contoured at 1.5 σ. (B) Fo-Fc density contoured at 3.0 σ in the same region calculated from the dataset for the GPP-bound structure. (TIF) [file pbio.1001911.s010.tif]
