# Supplementary material for: Structure of a Membrane-Embedded Prenyltransferase Homologous to UBIAD1
Source: PLoS Biol. 2014 Jul 22;12(7):e1001911. doi: 10.1371/journal.pbio.1001911 (PMC4106721; doi:10.1371/journal.pbio.1001911)
Supplement: Table S1 — Data collection and refinement statistics. (DOC) [file pbio.1001911.s011.doc]

|  | SeMet AfUbiA | Native AfUbiA/Mg^2+^/GPP | Native AfUbiA/Mg^2+^/DMAPP | Native AfUbiA/Cd^2+^ |
| --- | --- | --- | --- | --- |
| Data collection |  |  |  |  |
| Space group | P3_1_12 | P2_1_ | P2_1_ | C222_1_ |
| Cell Dimensions |  |  |  |  |
| a, b, c (Å) | 61.9, 61.9, 248.8 | 54.6, 218.9, 65.3 | 54.4, 218.5, 65.0 | 52.9, 89.9, 341.2 |
| ß (º) |  | 90.1 | 90.2 |  |
| Resolution (Å) | 3.2 (3.26-3.20)* | 2.40 (2.44-2.40) | 2.50 (2.54-2.50) | 3.07 (3.23-3.07) |
| R_sym_ or R_merge_ | 0.128 (>1) | 0.101 (0.488) | 0.116 (0.672) | 0.043 (0.643) |
| I/σ(I) | 27.7 (1.7) | 8.7 (2.3) | 9.1 (1.5) | 14.3 (1.1) |
| Completeness (%) | 99.9 (98.7) | 92.6 (91.7) | 99.2 (98.6) | 98.9 (98.9) |
|  |  |  |  |  |
| Refinement |  |  |  |  |
| Resolution (Å) | 3.20 (3.30-3.20) | 2.41 (2.45-2.41) | 2.50 (2.55-2.50) | 3.07 (3.26-3.07) |
| No. reflections | 17481 (1422) | 54436 (2516) | 51753 (2442) | 15519 (2567) |
| Completeness (%) | 99.4 (97.5) | 92.2 (85.0) | 98.5 (85.0) | 98.3 (99.4) |
| R_work/_ R_free_ (%) | 25.1/28.9 | 22.1/25.5 | 21.5/25.5 | 26.5/30.1 |
| No. atoms |  |  |  |  |
| Protein | 2149 | 8876 | 9041 | 4048 |
| Ligand/ion | 20 | 84 | 64 | 8 |
| Solvent |  | 25 | 27 |  |
| B-factors |  |  |  |  |
| Protein | 95.7 | 44.7 | 51.3 | 100.7 |
| Ligand/ion | 108.6 | 45.6 | 58.2 | 157.5 |
| Solvent |  | 38.7 | 48.3 |  |
| R.m.s deviations |  |  |  |  |
| Bond lengths (Å) | 0.006 | 0.008 | 0.008 | 0.009 |
| Bond angles (°) | 0.952 | 1.190 | 1.167 | 1.474 |
| PDB accession # | 4TQ5 | 4TQ3 | 4TQ4 | 4TQ6 |
