# Supplementary material for: Structure of a Membrane-Embedded Prenyltransferase Homologous to UBIAD1
Source: PLoS Biol. 2014 Jul 22;12(7):e1001911. doi: 10.1371/journal.pbio.1001911 (PMC4106721; doi:10.1371/journal.pbio.1001911)
Supplement: Table S2 — Complementation of menA− E. coli by WT and mutant EcMenA. OD600 measurements from three experiments of E. coli cultures grown under anaerobic conditions, used to calculate the bar graph in Figure 5C. (DOC) [file pbio.1001911.s012.doc]

|  | Exp. 1 | Exp. 2 | Exp. 3 | Mean | St. Dev. |
| --- | --- | --- | --- | --- | --- |
| WT | 0.47 | 0.44 | 0.47 | 0.46 | 0.017 |
| TrkH (-) | 0.014 | 0.03 | 0.028 | 0.024 | 0.0087 |
| N64A | 0.017 | 0.012 | 0.011 | 0.013 | 0.0032 |
| D68A | 0.016 | 0.01 | 0.014 | 0.013 | 0.0031 |
| D204A | 0.021 | 0.026 | 0.024 | 0.024 | 0.0025 |
| D208A | 0.019 | 0.031 | 0.037 | 0.029 | 0.0092 |
| Y141L | 0.034 | 0.022 | 0.025 | 0.027 | 0.0062 |
