# Supplementary material for: Structure of a Membrane-Embedded Prenyltransferase Homologous to UBIAD1
Source: PLoS Biol. 2014 Jul 22;12(7):e1001911. doi: 10.1371/journal.pbio.1001911 (PMC4106721; doi:10.1371/journal.pbio.1001911)
Supplement: Table S3 — Prenyltransferase activities of WT and mutant EcMenA. Normalized activities from three experiments with WT and mutant EcMenA, used to calculate the bar graph in Figure 5D. (DOC) [file pbio.1001911.s013.doc]

|  | Exp. 1 | Exp. 2 | Exp. 3 | Mean | St. Dev. |
| --- | --- | --- | --- | --- | --- |
| WT | 96.7 | 100 | 103 | 100 | 3.08 |
| EcUbiA (-) | 0.261 | 0.176 | 0.213 | 0.217 | 0.0427 |
| N64A | 0.54 | 0.571 | 0.632 | 0.581 | 0.0464 |
| D68A | 1.64 | 1.94 | 1.46 | 1.68 | 0.245 |
| D204A | 0.383 | 0.334 | 0.431 | 0.383 | 0.0486 |
| D208A | 0.256 | 0.395 | 0.279 | 0.310 | 0.0746 |
| Y141L | 0.279 | 0.437 | 0.425 | 0.381 | 0.0879 |
